# Editorial: Surgical treatment of thymic epithelial tumor and myasthenia gravis

**DOI:** 10.3389/fsurg.2025.1553723

**Published:** 2025-01-23

**Authors:** Piergiorgio Muriana

**Affiliations:** Department of Thoracic Surgery, IRCCS San Raffaele Scientific Institute, Milan, Italy

**Keywords:** thymoma, thymic carcinoma, thymic epithelial tumors, myasthenia gravis, surgery

**Editorial on the Research Topic**
Surgical treatment of thymic epithelial tumor and myasthenia gravis

The interaction between thymic epithelial tumors (TETs) and myasthenia gravis (MG) represents a fascinating and complex field in oncology, neuroscience, and thoracic surgery. Advances in minimally invasive surgery (MIS) and an evolving understanding of MG's pathophysiology have significantly improved patient care. TETs, the most common anterior mediastinal tumors, highlight the importance of surgery in achieving curative outcomes, especially in early-stage disease. Likewise, MG is commonly managed through thymectomy to alleviate symptoms and influence disease progression. Despite these advances, critical questions remain, necessitating further exploration of evolving strategies and challenges.

Surgical resection is the cornerstone treatment for TETs, providing excellent long-term outcomes despite challenges like large tumor size and mediastinal invasion even at the presentation of the disease. A meta-analysis demonstrated pooled 5-year and 10-year overall survival rates of 84% and 73%, respectively, across over 11,000 patients (1). Among all the prognostic factors, complete resection (R0) significantly improves prognosis, even in advanced stages, highlighting its importance.

The multicenter randomized Thymectomy Trial in Non-Thymomatous Myasthenia Gravis Patients Receiving Prednisone Therapy (MGTX) by Wolfe et al. was pivotal in establishing thymectomy's efficacy for non-thymomatous generalized MG (2). This randomized study revealed that extended transsternal thymectomy combined with prednisone led to superior outcomes compared to steroid therapy alone, including lower Quantitative Myasthenia Gravis (QMG) scores, reduced prednisone and azathioprine requirements, and fewer readmission for disease exacerbations. In a later report, follow-up confirmed these benefits over five years (3). The findings underscored thymectomy's durable benefits in managing MG without raising new safety concerns.

Recently, safety of thymectomy was questioned by the study by Kooshesh et al, who presented evidence of potential long-term risks associated with the surgical removal of thymus (4). In this study, thymectomy increased all-cause mortality—with a relative risk equal to 2.9—and a two-fold risk of cancer incidence within five years compared to controls who underwent cardiothoracic surgery without thymectomy. Additionally, thymectomy patients showed increased risk of post-operative autoimmune disorder development and higher overall and cancer-related mortality at long-term follow-up than in the general population. These findings highlight the need for cautious, individualized approaches when considering thymectomy.

This collection “Surgical Treatment of Thymic Epithelial Tumor and Myasthenia Gravis” explores topics ranging from the efficacy of MIS, including robotic-assisted and subxiphoid approaches, compared with traditional open surgery to the multimodal management of aggressive disease. By examining these emerging trends, this special issue encourages further research and collaboration among thoracic surgeons, neurologists, and oncologists.

The review articles by Alcasid et al. and Özçıbık Işık et al. agree on the comparable efficacy of open and minimally invasive approaches, such as robotic-assisted thoracic surgery (RATS) and video-assisted thoracic surgery (VATS), in achieving R0 resection, underscoring the effectiveness of extended thymectomy as the gold standard for both oncological and autoimmune outcomes. Moreover, MIS offers reduced postoperative morbidity, shorter hospital stays, and faster recovery, especially in early-stage or less invasive cases. Regarding advanced disease, according to Özçıbık Işık et al., surgical outcomes for stage IVA tumors are acceptable, but stage IVB requires multidisciplinary decision-making due to limited data. Again, R0 resection is critical, with re-operative surgery providing survival benefits in recurrent pleural metastases or localized recurrences.

The study by Kaba et al. supports the consensus that MIS is effective for early-stage TETs and MG treatment, demonstrating excellent long-term outcomes in 59 patients operated for stage I-II thymoma (58 by VATS and one by RATS), with 10-year disease-free survival rates of 96.6% and overall survival of 86.4%. Consistently with Alcasid and Özçıbık Işık, their findings underscore the importance of adhering to oncological principles, such as achieving R0 resection, for ensuring success. The authors emphasize the critical role of the surgeon's expertise in achieving favorable outcomes. While these approaches are optimal for early-stage cases, the exclusion of advanced-stage tumors highlights the need for tailored strategies based on individual tumor characteristics, disease stage, and even the surgical center expertise.

Leng et al. compared subxiphoid (SA) and lateral intercostal (LA) VATS approaches in resecting anterior mediastinal masses. The SA approach was later introduced in the clinical practice as an alternative to LA approach for the treatment of large masses providing superior exposure. Their retrospective analysis of 91 patients shows that, in two homogeneous groups, the LA approach offered shorter operative times, reduced hospital stays, and lower costs, making it an efficient choice for early-stage tumors. Both approaches had comparable safety profiles and low complication rates, though the steep learning curve of SA might explain its longer operative times and higher costs. The authors conclude that the choice of approach should consider tumor characteristics and patient-specific factors.

Meng et al. present a case report of multimodal treatment in a patient with unresectable thymic carcinoma, highlighting the role of immunotherapy in managing refractory disease. Their use of sintilimab, a PD-1 inhibitor, achieved prolonged partial remission after unresponsiveness to traditional treatments, underscoring the potential of immunotherapy for advanced or unresectable disease. This aligns with the broader shift towards personalized treatment strategies and innovative systemic therapies, particularly for advanced cases.

These studies collectively emphasize a nuanced approach to managing TETs and MG. MIS continue to demonstrate significant benefits for early-stage disease, while systemic therapies, such as immunotherapy, are increasingly critical for advanced or recurrent cases. The integration of surgical expertise with novel systemic approaches underscores the importance of interdisciplinary collaboration. Tailored treatment strategies, guided by tumor characteristics and and patient factors, remain essential for optimizing outcomes and advancing care in this complex field.
